# Association between community violence exposure and teen parental firearm ownership: data from a nationally representative study

**DOI:** 10.1186/s40621-024-00542-0

**Published:** 2024-11-14

**Authors:** Karissa R. Pelletier, Jesenia M. Pizarro, Regina Royan, Rebeccah Sokol, Rebecca M. Cunningham, Marc A. Zimmerman, Patrick M. Carter

**Affiliations:** 1 Center for Behavioral Health and Justice, School of Social Work, Wayne State University, 5447 Woodward Ave, Detroit, MI 48202 USA; 2 School of Criminology and Criminal Justice, Arizona State University, 411 North Central Ave Suite 600, Phoenix, AZ 85004 USA; 3 Department of Emergency Medicine, University of Michigan Medical School, 1500 E Medical Center Drive, Ann Arbor, MI 48109 USA; 4 Institute for Firearm Injury Prevention, University of Michigan, 1109 Geddes Avenue, Ann Arbor, MI 48109 USA; 5 School of Social Work, University of Michigan, 1080 S University Ave, Ann Arbor, MI 48109 USA; 6 Univ. of Michigan Injury Prevention Center, Univ. of Michigan Medical School, 2800 Plymouth Road, NCRC 10-G080, Ann Arbor, MI 48109 USA; 7 Youth Violence Prevention Center, Univ. of Michigan School of Public Health, 1415 Washington Heights, Ann Arbor, MI 48109 USA; 8 Dept of Health Behavior & Health Education, Univ of Michigan School of Public Health, 1415 Washington Heights 3790A SPH I, Ann Arbor, MI 48109 USA; 9 University of Minnesota, 202 Morrill Hall, 100 Church Street S.E., Minneapolis, MN 55455 USA

**Keywords:** Community violence exposure, Firearm ownership, Parents, Caregivers, Firearm violence

## Abstract

**Background:**

Firearm injuries are the leading cause of death for U.S. adolescents. Given the prevalence of firearm ownership in the U.S., particularly among parental figures in homes with children and teens, and the relationship between firearm access and injury outcomes, it is vital to shed light on potential parental motivations for keeping firearms in their homes. The purpose of this analysis was to examine whether exposure to community violence is associated with parental firearm ownership.

**Methods:**

Data from the Firearm Safety Among Children and Teens Consortium’s National Survey (6/24/2020-7/24/2020) was examined. The survey sample comprised parents/caregivers of high-school-age teens (age 14–18). The survey examined various measures, including firearm ownership, storage, community violence exposure, and sociodemographic characteristics. Stepwise logistic regression was used to examine the association between community violence exposure and parental firearm ownership.

**Results:**

The study included 2,924 participants, with 45.1% identifying as male, 12.9% identifying as Hispanic, and 25.3% identifying as non-White. Among these participants, 43.1% reported firearm ownership, and 49.9% reported exposure to community violence. Regression models demonstrate that community violence exposure is associated with an increased likelihood of firearm ownership among parents/caregivers of high-school age teens (OR = 1.08, *p* < 0.05). Other significant predictors of firearm ownership among parents/caregivers included parent/caregiver age (OR = 0.99, *p* < 0.01), marital status (OR = 1.29, *p* < 0.05), and educational attainment (OR = 0.60, *p* < 0.001).

**Conclusions:**

The findings supported the hypothesis that community violence exposure was associated with an increased likelihood of parental firearm ownership, even after adjusting for potential confounders. These findings contribute to the existing literature by shedding light on the possible contributing factors for firearm ownership among parents/caregivers of teens. Public health interventions focused on raising awareness about the risks of firearm access in households with youths, providing counseling on locked storage practices, and offering resources for accessing secure firearm storage options, such as rapid access storage, may contribute to reducing firearm access among youth. Additionally, community-based initiatives focused on violence prevention and addressing the root causes of community violence can help create safer environments, thereby reducing the perceived need for accessible firearms in the home by parents and caregivers.

**Supplementary Information:**

The online version contains supplementary material available at 10.1186/s40621-024-00542-0.

## Background

Firearms are the leading cause of death for U.S. high-school aged teens (Goldstick et al. 2021), with 93.1% of homicides and 49.4% of suicides among 14- to 18-year-olds in 2021 resulting from firearm injuries (CDC National Center for Injury Prevention and Control 2024). Between 2010 and 2019, an estimated 93,938 U.S. emergency department visits were for nonfatal firearm injuries among youth aged 14 to 18 (CDC National Center for Injury Prevention and Control 2003; Patel et al. 2021). Firearm injuries are associated with multiple long-term consequences (CDC National Center for Injury Prevention and Control 2003), including elevated rates of physical and mental health issues. Such injuries incur ongoing costs for affected individuals, their surrounding family members, and the healthcare system (Pulcini et al. 2021a, 2021b; Committee on Injury and Poison Prevention, American Academy of Pediatrics 2000). The healthcare expenses, law enforcement resources, legal proceedings, and lost productivity associated with firearm-related incidents amount to a substantial burden on communities and the overall society. It is estimated to be around $493.2 billion per year in 2020 (McLoughlin et al. 2019). Scholars examining firearm fatal and nonfatal injuries have also identified multiple disparities across age, gender, race, ethnicity, and sexual orientation/gender identity groups (Fowler et al. 2017). For example, most firearm-related suicides and homicides among youth occur among males (89% and 87%, respectively) (CDC National Center for Injury Prevention and Control 2003). Firearm homicide rates among black youth are two to four times higher than their Hispanic and Indigenous peers and ten times higher than white and Asian youth (Fowler et al. 2017). These disparities and the significant societal costs underscore the urgency of understanding factors, such as parental firearm ownership, that may be influenced by community violence exposure.

Across all injury intents, firearm access by high-school age teens remains the single most significant modifiable risk factor for all types of adolescent firearm injuries. Miller and Azrael (2022) found that 7 million more children lived in a home with a firearm than in previous years, and about 15% of firearms were stored unlocked and loaded (Lee et al. 2014; Miller et al. 2022). Scholars have also identified that unsecured firearm access in the home increases the risk of firearm homicide and suicide (Seewald et al. 2022). Taken together, this suggests that there are risks in keeping firearms in homes with adolescents, especially if they are stored, unlocked, and loaded.

While researchers have demonstrated the nature of the reciprocal relationship between community violence exposure and firearm carriage among youths and young adults (Chavez et al. 2022; Sokol et al. 2021), researchers have not examined the relationship between community violence exposure and parental firearm ownership. In this study, we addressed this gap in the literature by examining the relationship between community violence exposure and parental firearm ownership among a nationally representative sample of parents/caregivers of high-school age teens. We hypothesized that after controlling for sociodemographic factors and individual parental exposure to violence, community violence exposure is related to an increased likelihood of firearm ownership.

## Methods

Data are from the Firearm Safety Among Children and Teens (FACTS) Consortium’s cross-sectional web-based Gallop survey of 2,924 parents and primary caregivers of high-school teens (age 14–18). The survey was conducted from June to July 2020, with participants recruited from the Gallop Panel, a probability-based panel of non-institutionalized citizens constructed to represent the overall U.S. population. Gallop regularly recruits panel participants using address-based sampling and random-digit dialing methods encompassing landline and cellphone numbers. It intentionally oversamples young adults (age 18–34), those from lower educational backgrounds, and those from under-represented racial/ethnic groups. For this study, eligible panel participants included parents and primary caregivers for high-school age teens (age 14–18), defined to include both biological parents and other primary caregivers (e.g., grandparents) that reside with the teen at least some of the time. Known parents and caregivers were randomly sampled from the panel, and if they were found to have children aged 14–18, they were emailed invitations to participate in the study. Following informed consent, panelists self-administered the web-based survey (approximately 15 min). Remuneration was $5 for survey completion. The University of Michigan (U.M.) and Gallop IRBs approved all study procedures. This analysis focused only on the adult Parent/Caregiver sample.

### Measures

*Parental Firearm Ownership*: The dependent variable for this analysis was parental firearm ownership (yes/no). Ownership was measured using a single item (“Do you personally own a gun?”) adapted from the 2015 National Firearm Survey (NFS) (Sokol et al. 2022; Azrael et al. 2017).

*Community Violence Exposure*: Parent/caregivers perceptions of community violence exposure, the primary independent variable, were assessed using a 9-item version of the “Things I have Seen/Heard” Survey (Richters and Martinez 1990), measuring past 12-month exposure to a series of violent behaviors (e.g., hearing gunshots, seeing drug deals, seeing someone get shot) in their neighborhood. The standard response scale was modified to a dichotomous response (yes/no) for each item. A summary score (range 0–9) was created for analysis.

*Control Variables*: The Gallup survey collected data on various sociodemographic characteristics, including age, biological sex, race and ethnicity, region, educational attainment, marital status, and military service. For analyses, race and ethnicity was categorized as non-Hispanic White or non-White and/or Hispanic, and educational attainment was grouped into those with or without post-secondary education. This decision was made due to the small number of participants in individual racial, ethnic, and educational attainment categories, which did not allow for stable estimates when examining groups separately. While this approach was necessary to preserve statistical power, we acknowledge that it may obscure differences among racial minority, ethnic minority, and educational attainment populations.

Additionally, respondents were queried about the presence of young children (under the age of 10) in their household, in addition to teenagers, using questions derived from the 2016 American Community Survey. To assess the prevalence/frequency of partner (e.g., spouse, ex-spouse, dating partner) and non-partner (e.g., peers, friends, strangers, colleagues) victimization among various relationships, we used an adapted 9-item version of the Conflict Tactics Scale (CTS-2) (Straus et al. 1996), which captured incidents of moderate (e.g., pushed, shoved) and severe (e.g., hit, punched, used a knife/gun) violent behaviors. The adapted scale measured both victimization (i.e., actions done to the respondent) and violence (i.e., actions done by the respondent) on a response scale ranging from 0 (never) to 6 (> 20 times). These variables were then used to create a standard summary score by centering the variable using the mean and sum. Sup­plementary Material 1: Table S1 illustrates the coding schema for all variables.

### Statistical analysis

We analyzed the data with SPSS software at the *p*< 0.05 significance level. We weighted all descriptive statistics and analyses to be nationally representative of parents/caregivers with teenage children. Bivariate and multivariable analyses comparing the independent community violence exposure, and the dependent variable parental firearm ownership were first examined among the entire sample. Stepwise regression modeling was used as it allows for clarity at multiple stages and simultaneous estimation effects at multiple level (Hidalgo et al. 2013; Woltman et al. 2012). The stepwise regression modeling was completed with 2,380 parents/caregivers from the sample (*N*= 2,924) due to incomplete responses and the nature of the modeling. To elucidate how such factors may increase the likelihood of parental firearm ownership (Johnson et al. 2004; Carter et al. 2017; Branas et al. 2009), we ran four models: Model 1 examined demographic variables and other control variables, marital status, and education; Model 2 introduced parent/caregiver-partner and non-partner victimization; Model 3 introduced parent/caregiver community violence exposure. To examine the relationship between the control variables of biological sex, age, and education level, interaction terms between community violence exposure and each of those variables were created, respectively. Model 4 introduced the interaction between biological sex and community violence exposure. These interactions enabled us to examine whether the relationship between community violence exposure and parental firearm ownership changes dependent on the sex of the parental respondant (Pelletier and Pizarro 2019).

## Results

Table 1 provides the descriptive statistics for this analysis. Among all sampled households in this analysis, 43.1% (*n* = 1,261) reported firearm ownership. Most firearm owners were non-White and/or Hispanic, married, male, had completed some college/trade school (or beyond), and had an average age of 47.1 years old. Table 2 provides the types of community violence exposure within the sample. In total, 49.9% (*N* = 1,425) of the sample experienced at least one form of community violence exposure, with the majority indicating they had either heard gunshots (24.0%, *N* = 703) and/or had observed someone be arrested (9.0%, *N* = 264). For additional percentages and the original count of those who endorsed each of the community violence variables, please see Table 2. Supplementary Material 1: Table S2 shows the counts of partner and non-partner victimization counts. Approximately 3.3% (*N* = 95) of the sample reported experiencing partner victimization at least once, and about 1.8% (*N* = 53) of the sample reported experiencing non-partner victimization at least once in the 12 months prior to the survey.

**Table 1:** Descriptive statistics for victimization and demographic variables for parent firearm ownership, *N* = 2,924

	Parent has a firearm in the house		Chi-Squares
	No *N* (%)	Yes *N* (%)	
Sex			273.45***
Male	503 (39.1)	785 (60.9)	
Race			14.39***
Non-white and/or Hispanic	449 (28.0)	274 (21.8)	
Marital status			12.20***
Married	1,308 (54.5)	1,093 (45.5)	
Education			46.11***
Some college or trade school and beyond	1,209 (75.6)	803 (63.9)	
Age (mean, stand. dev.)	47.1 (9.4)	47.8 (10.2)	--
Parent partner victimization (mean, stand. dev.)	0.15 (0.8)	0.06 (0.4)	--
Parent non-partner victimization (mean, stand. dev.)	0.07 (0.5)	0.04 (0.5)	--

***p < 0.001

**Table 2:** Descriptive findings regarding community violence exposure among a nationally representative sample of parents of high-school age teens (age 14–18), *N* = 2,924

	% (*N*)*	Number of parents who endorsed community violence exposure
Heard guns being shot	24.0 (703)	1,158
Have seen somebody arrested	9.0 (264)	552
Have seen drug dealing	8.2 (241)	363
Have seen somebody get beaten up	2.8 (83)	154
Have seen somebody get stabbed	0.3 (9)	59
Have seen someone pull a knife on another person	0.8 (23)	61
Have seen someone pull a gun on another person	0.9 (27)	59
Have seen someone get shot	0.7 (21)	31
My house has been broken into	1.5 (55)	55

*Note: 50.1% (*N* = 1,466) did not report experiencing any community violence exposure and 33 were missing data

The multivariate logistic regression analyses demonstrated that community violence exposure was associated with an increased likelihood of parental firearm ownership (Table 3). Model 1 examined demographic and other control variables, marital status, and education. Parent/caregiver sex, age, marital status, and level of education emerged as significant in this Model, suggesting that younger parents/caregivers (Odds Ratio (OR) = 0.99, *p* < 0.01) and parents/caregivers with less education (OR = 0.60, *p* < 0.001) had a decreased likelihood of owning a firearm, while households with married parents/caregivers (OR = 1.29, *p* < 0.05) were at an increased likelihood. Model 2 introduced parent/caregiver-partner and non-partner victimization. In this Model, we found that parent/caregiver-partner and non-partner victimization were not significantly associated with parental firearm ownership. However, the sociodemographic variables remained significant. Model 3 introduced parent/caregiver community violence exposure. In this Model, we found that exposure to community violence was significantly associated with an increased likelihood of parental firearm ownership (OR = 1.08, *p* < 0.05). Finally, model 4 introduced the interaction between sex and community violence exposure. The results of Model 4 suggested that an interaction between community violence exposure and sex does increase the likelihood of firearm ownership (OR = 1.42, *p* < 0.001), such that increased levels of community violence exposure among males is associated with greater levels of firearm ownership. In addition to analyzing community violence exposure as a continuous variable, we examined it as a dichotomous variable (any exposure vs. no exposure). Our analysis revealed that parental firearm ownership remains significantly associated with any exposure to community violence, with an odds ratio of OR = 0.09, *p* < 0.05.

**Table 3:** Hierarchical logistic regression for exposure to community violence, victimization, and Demographic Variables for Parent gun ownership, *N* = 2,380

Covariates	Model 1		Model 2		Model 3		Model 4	
	β	OR	β	OR	β	OR	β	OR
*Sociodemographic variables*								
Sex: Male	1.27***	3.55	1.26***	3.52	1.25***	3.50	0.98***	2.66
Race and ethnicity: Non-white and/or Hispanic	– 0.01	0.99	– 0.05	0.95	– 0.06	0.95	– 0.05	0.96
Age	– 0.02***	0.98	– 0.02**	0.98	– 0.02**	0.99	– 0.02**	0.99
Marital status: Married	0.25	1.29	0.29*	1.33	0.28*	1.33	0.26*	1.29
Education: High school or Below	– 0.54***	0.58	– 0.52***	0.59	– 0.50***	0.61	– 0.52***	0.60
*Violence exposure and victimization variables*								
Parent partner victimization	--	--	0.12	1.13	0.12	1.13	0.10	1.10
Parent non-partner victimization	--	--	0.06	1.06	0.06	1.06	0.04	1.04
Parent community violence exposure	--	--	--	--	0.07*	1.08	0.08	1.09
*Interaction*								
Interaction between sex and community violence exposure	--	--	--	--	--	--	0.35***	1.42
Constant	0.28	1.33	0.06	1.06	0.10	1.10	0.21	1.24

Note: *p $\:\le\:$ 0.05; **p $\:\le\:\:$0.01; ***p $\:\le\:\:$0.001; Model chi-square: $\:\chi^2$ (12) = 276.83, *p* < 0.001

## Discussion

This study examined the association of community violence exposure to firearm ownership among parents and caregivers of high-school age adolescents. Using multivariable analyses, we found that exposure to community violence was significantly associated with an increased likelihood of parental firearm ownership, even after controlling for potential confounding variables. Although we did not directly measure the motivations for firearm ownership, prior literature suggests that community violence, including vicarious exposure, may influence parents’/caregivers’ decisions to own firearms as a perceived protective measure (Sokol et al. 2021, 2022; Carter et al. 2020). This finding underscores the complex interplay between perceived risk and protective behaviors among parents and caregivers, particularly in environments where violence is prevalent. Hence, scholars should also consider non-direct personal exposures in future studies.

Moreover, the study highlights the importance of demographic factors in shaping parental firearm ownership patterns. While we did not explicitly measure risk perception, prior research suggests that younger parents/caregivers and those with lower education levels may perceive different risks or have different reasons for not owning firearms. Conversely, households with married parents/caregivers were more likely to own firearms, which could suggest that family dynamics or shared decision-making processes may play a role in firearm ownership practices, though this remains speculative and warrants further exploration.

The identification of significant interactions between sex and community violence exposure further demonstrated the nuanced nature of firearm ownership dynamics. The results revealed that male parents/caregivers were more likely to own firearms, and the association between community violence exposure and firearm ownership was stronger among men. This finding is consistent with prior studies that suggest men may be more likely to own firearms due to perceptions of protection or adherence to traditional gender norms, though we caution that our data do not directly address these motivations (Carlson 2015; Stroebe et al. 2017; Metzl and MacLeish 2015). Further research should investigate the specific factors driving these gendered differences in firearm ownership.

Interestingly, although exposure to community violence emerged as significant, prior partner and non-partner victimization did not. This unexpected finding might be due to lower levels of prior victimization among the entire sample. Indeed, prior research examining the effect of prior victimization on the increased likelihood of firearm carriage and ownership has focused on individuals with elevated rates of prior victimization (Pizarro et al. 2021; Bostwick 2023). This discrepancy suggests that future research should examine victimization more closely, especially among populations with higher exposure rates, to fully understand its relationship with firearm ownership.

While there has been a linkage between firearm carriage and increased likelihood of serious injury and death (Leppink 2018) as well as firearm possession and community violence exposure (Sokol et al. 2021), to the best of our knowledge, this was the first study to examine the association of community violence exposure with parental firearm household ownership. Our findings contribute to the growing body of research on the complex relationship between community violence and firearm ownership, though additional studies are needed to verify and expand upon these results. The findings underscored the multifaceted nature of firearm ownership dynamics within the context of community violence, highlighting significant implications for public health and policy interventions. Targeted interventions addressing community violence prevention and firearm safety education are warranted to mitigate the risk of firearm-related injuries among high-school age teens.

Moreover, efforts to promote locked storage practices and firearm safety policies (e.g., child access prevention laws) are essential to prevent unauthorized access to firearms among adolescents. While our study did not directly assess firearm storage behaviors, these recommendations are based on well-documented evidence linking firearm access to youth safety risks. For parents/caregivers with firearms in the home, as suggested by Sokol and colleagues (Chavez et al. 2022), intervention strategies should include activities that reinforce personal values of family safety and protection along with safe firearm storage. Emphasizing the importance of locked firearm storage to reduce youth access is a key prevention strategy, particularly in light of prior research linking firearm carriage and access to violent injury and death (Lee et al. 2014; Miller and Azrael 2022; Seewald et al. 2022). Recent scholars have found that personalized secure storage devices and other new technology provide promise for firearm owners to have rapid access to their firearms while also preventing adolescents from accessing these guns (Carter et al. 2020; Nehra et al. 2021).

Finally, given the relationship between community violence exposure and firearm use, this study also underscored the need for comprehensive approaches to address the underlying violence driving a perceived need for maintaining firearms for protection, including socio-economic disparities and community-level factors contributing to violence (Miller et al. 2001). Such upstream-focused community-level interventions have been shown to reduce community violence in areas with high levels of violence. Additionally, intervention and prevention strategies should include individual-level programs addressing violence reduction, such as conflict resolution and investment in academic programs, given the substantial overlap between general violence and community violence (Kondo et al. 2018).

## Limitations

Despite these findings, it is not without several limitations. First, it is essential to note that this study focused on household that have children aged 14–18. However, while this is a limitation, our study does speak to the broad majority of adolescents who would be most at risk for intentional firearm use and injury (Goldstick et al. 2021). Second, this study focused on reported parental firearm ownership. We decided not to include broader measures of household firearm ownership given known issues with the reliability of individuals assessing other household members’ firearm (Ali-Saleh Darawshy et al. 2020) ownership behaviors. Finally, the survey data was self-reported, which may introduce the possibility of social desirability and recall bias in participants. As these are important limitations, particularly in studies relying on self-reported data, they may affect the accuracy of responses regarding past violence exposure and firearm ownership. Social desirability bias could lead respondents to underreport sensitive behaviors, such as firearm ownership, while recall inaccuracy might affect the reporting of past experiences of violence. While this presents the possibility for social desirability and/or recall bias, prior studies have demonstrated that these self-report measures, such as the Gallup survey used here, are reliable and valid, particularly when confidentiality and privacy are assured, as was the case in this study (Schell et al. 2020; Tourangeau and Yan 2007; Brener et al. 2003; Kreuter et al. 2008).

## Conclusions

In conclusion, firearms have been the leading mechanism for death for 0- to 19-year-olds. Thus, it is imperative to understand the different factors that can lead to firearm access, carriage, and possession. This nationally representative survey study of parents/caregivers and teens sheds light on the reasons for firearm possession in the home. As indicated by the findings, violence exposure had a significant impact on parental firearm possession, and addressing such community-level factors is a necessary focus of multilevel interventions as this may be a salient factor influencing decisions about household firearm ownership, storage practices, and subsequent injury risk.

This study provides valuable insights into the complex interplay between community violence exposure and parental firearm ownership among households with high-school age teens. By elucidating the drivers of firearm ownership and the associated risk factors, this study provides policymakers and public health professionals knowledge that can be used to develop targeted interventions to promote firearm safety.

## Electronic supplementary material

Supplementary Material 1

## Data Availability

The data used in this study is available upon request from the Institute of Firearm Injury Prevention at the University of Michigan. The raw data will also be available in the University of Michigan’s data repository, which can be found here: https://www.openicpsr.org/openicpsr/facts-open.
