# Supplementary material for: Association between community violence exposure and teen parental firearm ownership: data from a nationally representative study
Source: Inj Epidemiol. 2024 Nov 14;11:64. doi: 10.1186/s40621-024-00542-0 (PMC11566730; doi:10.1186/s40621-024-00542-0)
Supplement: Supplementary file 1 — Supplementary Material 1 [file 40621_2024_542_MOESM1_ESM.docx]

| Supplementary Table 1. Coding Schema for Exposure to Community Violence, Victimization, and Demographic Variables for Parent Gun Ownership  N = 2,924 | |
| --- | --- |
| *Sociodemographic Variables* | |
| Sex | 0 = Female, 1 = Male |
| Race | 0 = White, not Hispanic; 1 = Hispanic and/or Non-White |
| Age | Continuous variable from ages 19 to 86 |
| Marital Status | 0 = Unmarried; 1 = Married |
| Education | 0 = High School Degree or Less; 1 = Some College or Trade School and Beyond |
| *Violence Exposure and Victimization Variables* | |
| Parent Community Violence Exposure | Scale variable ranging from 0 (no community violence exposure indicated) to 9 with the following community violence exposure variables: heard gun shots, seen someone get arrested, seen drug dealing, seen someone beat up, seen someone stabbed, seen someone pull a knife, seen someone pull a gun, seen someone get shot, and had house broken into |
| Parent Partner Victimization | Scale variable ranging from 0, or no partner victimization reported, to >20 incidents of partner victimization reported, used to create a standard summary score |
| Parent Non-Partner Victimization | Scale variable ranging from 0, or no non-partner victimization reported, to >20 incidents of non-partner victimization reported, used to create a standard summary score |

| Supplementary Table 2. Counts and Percentages for Parent/Caregiver Partner and Non-Partner Victimization  N = 2,924 | | |
| --- | --- | --- |
|  | N | % |
| Parent/Caregiver Partner Victimization |  |  |
| No victimization reported | 2,361 | 80.7 |
| One or more incidents of victimization reported | 95 | 3.3 |
| Missing/No response | 468 | 16.0 |
| Parent/Caregiver Non-Partner Victimization |  |  |
| No victimization reported | 2869 | 98.2 |
| One or more incidents of victimization reported | 53 | 1.8 |
| Missing/No response | 2 | 0.1 |
